# Improving bridge effect to overcome interspecific hybrid sterility by pyramiding hybrid sterile loci from *Oryza glaberrima*

**DOI:** 10.1038/s41598-023-49914-4

**Published:** 2023-12-27

**Authors:** Jing Li, Jiawu Zhou, Peng Xu, Ying Yang, Xianneng Deng, Wei Deng, Yu Zhang, Yonggang Lv, Qiuhong Pu, Dayun Tao

**Affiliations:** Yunnan Seed Laboratory / Yunnan Key Laboratory for Rice Genetic Improvement, Food Crops Research Institute, Yunnan Academy of Agricultural Sciences (YAAS), Kunming, 650200 People’s Republic of China

**Keywords:** Agricultural genetics, Plant breeding, Genetics

## Abstract

In order to evaluate the genetic effect caused by hybrid sterile loci, NILs with *O. glaberrima* fragment at six hybrid sterile loci under *O. sativa* genetic background (single-locus-NILs) were developed; two lines harboring two hybrid sterile loci, one line harboring three hybrid sterile loci were further developed. A total of nine NILs were used to test cross with *O. sativa* recurrent parent*,* and *O. glaberrima* accessions respectively. The results showed that the sterility of pollen grains in F_1_ hybrids deepened with the increase of the number of hybrid sterile loci, when the nine lines test crossed with *O. sativa* recurrent parent. The F_1_ hybrids were almost completely sterile when three hybrid sterile loci were heterozygeous. On the other hand, the single-locus-NILs had limited bridge effect on improving pollen grain fertility of interspecific hybrids. Compared single-locus-NILs, the multiple-loci-NILs showed increasing effect on pollen fertility when test crossing with *O. glaberrima* accessions. Further backcrossing can improve the fertility of pollen grain and spikelet of interspecific hybrids. The optimal solution to improve the fertility of interspecific hybrid can be utilization of pyramiding bridge parent plus backcrossing. This report has potential for understanding the nature of interspecific hybrid sterility, and overcoming the interspecific hybrid F_1_ pollen grain sterility between *O. sativa* and *O. glaberrima*.

## Introduction

Asian cultivated rice (*Oryza sativa* L.) is a prime food crop world-wide. However, advance in genetic improvement of rice has encountered problems owing to the narrow genetic diversity and the bottleneck of further yield increase^1^. African cultivated rice (*O. glaberrima* Steud.) is deemed to be a potential source of useful genes for improving Asian cultivated rice by hybridization as both cultivated species have the same AA genome and similar sequence arrangement^2,3^. However, there are strong reproductive barriers in the interspecific hybrids between the two cultivated species^4^. The F_1_ hybrids barely produce fertile pollen grain and as a result that the valuable genes are very difficult to be introgressed because of the strong hybrid sterility (HS). Therefore, HS is one of the main hindrances against the utilization of useful genes from the African cultivated rice for Asian cultivated rice improvement.

To date, at least 11 HS loci were reported as gamete eliminators or pollen killers between *O. sativa* and *O. glaberrima*^5^, and one of them were cloned^6–8^. The cumulative effects of these HS loci led to the complete male sterility in F_1_ hybrids of interspecific hybrid. *O. glaberrima* and *O. sativa* varieties possessed the genotype *S-g* and *S-s,* respectively, at the HS locus. Homozygotes of *S-s/S-s* and *S-g/S-g* show normal fertility, while *S-s* or *S-g* gametes are aborted when the sporophytic plants have the heterozygous genotype *S-s/S-g* in an *O. sativa* background. At the most HS loci reported, *S-s* gametes are aborted in the heterozygous genotype as the result the gametes of *O. glaberrima* are preferentially transmitted to the next generation and these HS loci were called African rice selfish loci. Recently, Feng et al.^9^ reported an Asian rice selfish locus *S58*, the *S58-g* gametes was aborted in the heterozygous genotype and caused a transmission advantage for the Asian rice allele of *S58* in the hybrid offspring.

Based on the known genetic information, if the HS loci in a given *O. sativa* background could be substituted by the neutral alleles or the corresponding alleles from *O. glaberrima*, it would be possible to overcome the interspecific HS between *O. sativa* and *O. glaberrima*^10,11^. It was reported that the *O. sativa* lines carrying the *S1-g* allele from *O. glaberrima* can be used as bridge parents to improve the fertility of hybrids between *O. glaberrima* and *O. sativa*^12,13^. However, the bridge effect of others HS loci and their pyramided lines remain unknown.

In our previous study, six HS loci, *S1, S19, S20, S37*(t)*, S38*(t)*, S39*(t) were identified from the crosses between *O. glaberrima* and *O. sativa*^14–16^*.* The *S1* and *S37*(t) loci functioned as the “gamete eliminator”: both male and female gametes carrying the allele of *O. sativa* were aborted in the heterozygotes. The *S19*, *S20, S38(t), S39(t)* locus functioned as the “pollen killer”: only male gametes carrying the allele of *O. sativa* were aborted in the heterozygotes.

In order to improve the bridge effect to overcome interspecific reproductive barrier, the near isogenic lines (NILs) carrying single and multiple O. *glaberrima* fragments at *S1, S19, S20, S37*(t), *S38*(t) and *S39*(t) were developed. The genetic effect of the HS loci were investigated in this study.

## Results

### Development of NILs harboring single and multiple *O. glaberrima* fragments at HS loci

**Figure 1:**
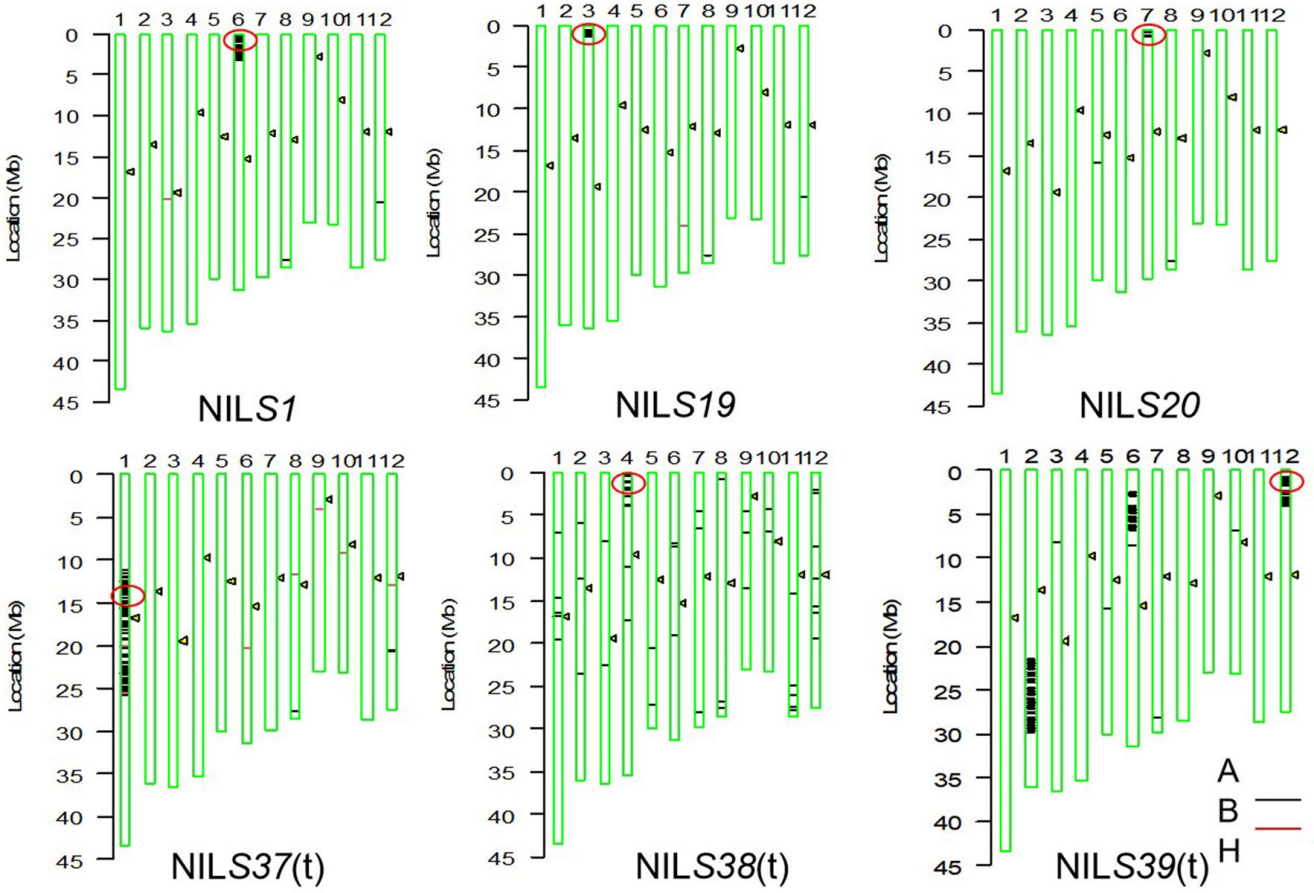
Genetic background screen of six NILs with single hybrid sterile allele from *O. glaberrima* using C6AIR. Twelve chromosomes of rice are labelled from 1 to 12 and the triangles indicated the positions of the centromere. The reference genome is *O. sativa* DJY1. The circle indicate the positions of the target locus. The black lines indicated the positions of the SNP with homozygous genotypes where genomic fragments of the donor parent were introgressed, red lines indicated the positions of the SNP with heterozygous genotypes, and the genotypes of the rest genomic regions were the same as the recurrent parent DJY1.

A genomics-based introgression of target single HS loci from the donors into DJY1 has been implemented using SSR markers. The high quality genotyping data for the candidate plants were provided on 6777 SNP markers. Six plants that carrying the target chromosomal fragment from the *O. glaberrima* accessions at HS loci of *S1*, *S19*, *S20*, *S37*(t)*, S38*(t) and *S39*(t) respectively and the genetic background were similar to the recurrent parent DJY1 were selected and denoted as single-loci-NILs, and named as NIL*S1*, NIL*S19*, NIL*S20*, NIL*S37*(t), NIL*S38*(t), NIL*S39*(t) respectively (Supplementary Table 1). Actually, the NILS*39*(t) harbored another two fragments from the donor parent and the genomic regions containing *S1*, *S37*(t), *S39*(t) had large linkage fragments from their donor parents, which may have potential adverse genetic effects (Fig. 1).

All the F_2_ self-pollinated plants showing the homozygous to *O. glaberrima* alleles on *S1* and *S37*(t) loci in the cross of NIL*S1*/NIL*S37*(t) because the *S1* and *S37*(t) loci functioned as the “gamete eliminator” and as the result only the gametes carrying the allele of *O. glaberrima* survived (Table 1). The pyramiding line combining two HS loci of *O. glaberrima* allele was denoted as two-loci-NIL, or multiple-loci-NIL, and named this line as NIL*S1S37*(t).

**Table 1:** Pollen grain and spikelet fertility, and genotypes of F_1_ plants in the crosses between DJY1 and the NILs.

Materials	Generation	Pollen grain fertility (%)	Spikelet fertility (%)	*S1* Chr.6^a^		*S19* Chr.3		*S20* Chr.7		*S37*(t) Chr.1		*S38*(t) Chr.4		*S39*(t) Chr.12	
				RM190	RM587	RM3372	RM22	RM20847	RM20852	RM449	RM513	RM16260	RM5414	RM1880	RM4
DJY1	P	99.05 ± 0.25	92.10 ± 3.07	s^b^	s	s	s	s	s	s	s	s	s	s	s
NIL*S1*	P	99.25 ± 0.31	91.19 ± 4.62	g	g	s	s	s	s	s	s	s	s	s	s
NIL*S19*	P	98.43 ± 1.46	86.42 ± 8.95	s	s	g	g	s	s	s	s	s	s	s	s
NIL*S20*	P	99.31 ± 0.29	93.12 ± 3.89	s	s	s	s	g	g	s	s	s	s	s	s
NIL*S37*(t)	P	98.84 ± 0.57	92.13 ± 3.77	s	s	s	s	s	s	g	g	s	s	s	s
NIL*S38*(t)	P	99.00 ± 0.42	90.11 ± 1.53	s	s	s	s	s	s	s	s	g	g	s	s
NIL*S39*(t)	P	97.63 ± 1.08	93.30 ± 5.64	s	s	s	s	s	s	s	s	s	s	g	g
NIL*S1S20*	P	99.06 ± 0.38	77.08 ± 5.00	g	g	s	s	g	g	s	s	s	s	s	s
NIL*S1S37*(t)	P	98.78 ± 0.54	78.75 ± 3.87	g	g	s	s	s	s	g	g	s	s	s	s
NIL*S1S20S37*(t)	P	98.93 ± 0.39	79.37 ± 4.43	g	g	s	s	g	g	g	g	s	s	s	s
DJY1/NIL*S1*^c^	F_1_	47.83 ± 1.85	51.97 ± 2.12	H	H	s	s	s	s	s	s	s	s	s	s
DJY1/NIL*S19*	F_1_	48.60 ± 3.56	89.96 ± 4.02	s	s	H	H	s	s	s	s	s	s	s	s
DJY1/NIL*S20*	F_1_	49.10 ± 4.07	88.40 ± 2.34	s	s	s	s	H	H	s	s	s	s	s	s
DJY1/NIL*S37*(t)	F_1_	58.91 ± 8.27	44.12 ± 1.32	s	s	s	s	s	s	H	H	s	s	s	s
DJY1/NIL*S38*(t)	F_1_	46.79 ± 1.51	82.65 ± 5.11	s	s	s	s	s	s	s	s	H	H	s	s
DJY1/NIL*S39*(t)	F_1_	47.32 ± 2.82	75.72 ± 12.71	s	s	s	s	s	s	s	s	s	s	H	H
DJY1/NIL*S1S20*	F_1_	33.81 ± 1.71	42.69 ± 9.30	H	H	s	s	H	H	s	s	s	s	s	s
DJY1/NIL*S1S37*(t)	F_1_	31.46 ± 10.0	50.19 ± 4.74	H	H	s	s	s	s	H	H	s	s	s	s
DJY1/NIL*S1S20S37*(t)	F_1_	3.35 (n = 1)	0.00	H	H	s	s	H	H	H	H	s	s	s	s

^a^Locus name and chromosome.

^b^s, H and g indicated DJY1-homozygous, heterozygous and *O. glaberrima*-homozygous genotypes, respectively.

^c^The symbol “/” indicated the hybridization event.

With the assistance of SSR markers linked to *S1* and *S20*, the F_2_ self-pollinated plants from the cross of NIL*S1*/NIL*S20* showing homozygous to *O. glaberrima* allele at the *S1* and *S20* loci were selected, then NIL*S1S20* was obtained. The selected plants with homozygous allele of *O. glaberrima* at the *S1* and *S20* loci also showed the normal pollen and spikelet fertility (Table 1).

To pyramid the three loci, *S1*, *S20* and *S37*(t) of *O. glaberrima* alleles in *O. sativa* genetic background, the SSR markers linked to them were used to genotype the F_2_ plants in the cross of NIL*S1*/NIL*S37*(t)//NIL*S20*. The plants homozygous to *O. glaberrima* allele at all three loci showing normal pollen and spikelet fertility were obtained (Table 1). The pyramiding line was designated as NIL*S1S20S37*(t)*.*

### Genetic effect caused by HS loci between *O. sativa* and* O. glaberrima*

For the single-locus-NILs, the pollen grain and spikelet fertility of F_1_ plants were semi-sterile in the crosses between DJY1 and NIL*S1*, NIL*S37*(t); the pollen grains were semi-sterile and the spikelet fertility was normal in the crosses between DJY1 and NIL*S19*, NIL*S20*, NIL*S38*(t), and NIL*S39*(t) (Table 1).

When the multiple-loci-NILs were crossed with DJY1, the fertility of pollen grain was significantly lower than those of the single-locus-NILs. The NIL*S1S20* and NIL*S1S37*(t) containing two HS loci caused about two-thirds of pollen abortion in hybrids and the F_1_ pollen grain fertility were 33.81% and 31.46%, respectively. In particular, the pollen grain of F_1_ plants was 3.35% in the cross between DJY1 and the NIL*S1S20S37*(t) (Table 1), which was the lowest among the test hybrids. These results showed that the cumulative effect of these HS loci in F_1_ hybrids was remarkable. When NIL*S1* and NIL*S37*(t) crossed with DJY1, the pollen and spikelet fertility of F_1_ plant were semi-fertility (Table 1) which followed the gamete eliminate model. However, the spikelet fertility of F_1_ plants was still semi-fertility (50.19%) when the NIL*S1S37*(t) was crossed with DJY1 (Table 1). It is unclear in regard to the interaction between these two loci.

### Bridge effect of single-locus-NILs and multiple-loci-NILs

There was no significant difference on pollen fertility between reciprocal F_1_s of all combinations (*P* = 0.94), indicated that no cytoplasmic effect was involved in hybrid sterility between the two *O. glaberrima* accessions and the *O. sativa* variety DJY1 (data not shown). The difference of F_1_ pollen grain fertility between the two interspecific crosses, DJY1 × IRGC102263 and DJY1 × IRGC103469 was not significant (*P* = 0.08), indicated that the compatibility between DJY1 and the two *O. glaberrima* accessions was consistent. The F_1_ pollen fertility data of reciprocal crosses between each NIL (or recurrent parent) and two African rice accessions was merged to obtain larger sample size.

The F_1_ progenies between the six single-locus-NILs and the *O. glaberrima* accessions showed poor pollen grain fertility although there were significant difference among them (Table 2), indicated that single-locus-NILs had limited bridge effect on improving pollen grain fertility of interspecific hybrids. Student's t test indicated that when NIL*S19*, NIL*S20*, and NIL*S37*(t) were crossed with the test *O. glaberrima* accessions, the average pollen fertility of F_1_ was 0.49%, 1.72%, 0.85%, respectively, which was notably higher than that of the control (the crosses between DJY1 and *O. glaberrima* accessions) (0.15%). Among them, *S20* locus had the largest effect on improving pollen grain fertility of interspecific F_1_ hybrids, followed by *S37*(t), and *S19* was only significant at 0.05 level. While the progenies derived from NIL*S1*, NIL*S38*(t) and NIL*S39*(t), were similar to the control.

**Table 2:** Pollen grain fertility of F_1_ hybrids from the crosses between two *O. glaberrima* accessions and NILs.

Crosses	Mean ± SD (%)	Least value (%)	Maximum value (%)	Plant number
DJY1/*O. glaberrima*^a^	0.15 ± 0.39	0.00	1.31	19
NIL*S1*/*O. glaberrima*	0.12 ± 0.25	0.00	0.72	13
NIL*S19*/*O. glaberrima*	0.49 ± 0.86*	0.00	2.05	7
NIL*S20*/*O. glaberrima*	1.72 ± 2.39**	0.00	8.42	20
NIL*S37*(t)/*O. glaberrima*	0.85 ± 0.94**	0.00	4.10	23
NIL*S38*(t)/*O. glaberrima*	0.00 ± 0.00	0.00	0.00	5
NIL*S39*(t)/*O. glaberrima*	0.10 ± 0.24	0.00	0.60	6
NIL*S1S20*/*O. glaberrima*	4.73 ± 3.11**	1.67	8.85	5
NIL*S1S37*(t)/*O. glaberrima*	5.96 ± 0.70**	5.46	6.45	2
NIL*S1S20S37*(t)/*O. glaberrima*	3.90 ± 2.10**	1.83	7.35	7

^a^The symbol “/” indicated the hybridization event.

*Means significant at 0.05 level; **means significant at 0.01 level.

The pollen grain fertility of F_1_ hybrids between the multiple-loci-NILs and *O. glaberrima* test accessions were notably higher than that of control (the crosses between DJY1 and *O. glaberrima* accessions). The F_1_ pollen fertility was 5.96% in the hybrids between NIL*S1S37*(t) and *O. glaberrima* accessions, and 4.73% in the hybrids between NIL*S1S20* and *O. glaberrima* accessions. The tri-loci line NIL*S1S20S37*(t) produced notably higher pollen fertility (3.90%) of F_1_ hybrids than that of single-locus-NILs. However, the F_1_ pollen fertility between the tri-loci-NIL and the test *O. glaberrima* accessions showed no significant difference from those of the F_1_ progenies between two-loci-NILs and test *O. galberrima* accessions (Table 3). Totally, multiple-loci-NILs showed higher bridge effect than the single-locus-NILs on overcoming interspecific hybrid sterility barriers between *O. sativa* and *O. glaberrima*. All of the F_1_ progenies from the crosses for bridge effect evaluation were complete spikelet sterility.

**Table 3:** Pollen grain fertility of BC_1_F_1_ from the crosses between NILs and two *O. glaberrima* accessions.

Crosses	Average (%)	Least value (%)	Maximum value (%)	Plant number
DJY1/*O. glaberrima*//DJY1^a^	5.06 ± 4.89	0.00	25.85	69
NIL*S1*/*O. glaberrima*//NIL*S1*	11.87 ± 15.65	0.00	85.81	207
NIL*S19*/*O. glaberrima*//NIL*S19*	9.87 ± 11.19	0.00	42.96	43
NIL*S20*/*O. glaberrima*//NIL*S20*	10.27 ± 11.41	0.00	55.08	98
NIL*S37*(t)/*O. glaberrima*//NIL*S37(t)*	8.14 ± 10.21	0.00	46.34	47
NIL*S38*(t)/*O. glaberrima*//NIL*S38*(t)	13.77 ± 18.27	0.00	53.69	14
NIL*S39*(t)/*O. glaberrima*//NIL*S39*(t)	8.36 ± 10.99	0.00	36.09	24
NIL*S1S20*/*O. glaberrima*//NIL*S1S20*	44.04 ± 26.60**	3.11	97.00	50
NIL*S1S37*(t)/*O. glaberrima*//NIL*S1S37*(t)	49.50 ± 25.90**	19.57	81.71	4

^a^The symbol “/” indicated the first hybridization event, and “//” indicated the second hybridization event.

*Means significant at 0.05 level; ** means significant at 0.01 level.

### Backcrossing can be used to improve pollen fertility of interspecific hybrids after the hybridization

Compared with the F_1_ generation, the average pollen grain fertility of BC_1_F_1_ generation increased significantly. The average pollen grain fertility of the BC_1_F_1_ individuals involving the six single-locus-NILs ranged from 8.14% to 13.77% while that of the control’s was 5.06%. The highest pollen grain fertility reached 85.81% in the BC_1_F_1_ individuals from the cross between NIL*S1* and *O. glaberrima*, which was more than three times of the control (Table 3). However, the average pollen grain fertility in the BC_1_F_1_ segregation population derived from the six single-locus-NILs did not notably differ from the control because of the large standard deviation value. When *O. glaberrima* accessions were test crossed with the two-loci-NILs, NIL*S1S20* and NIL*S1S37*(t), their average pollen grain fertility in the BC_1_F_1_ population was 44.04% and 49.50%, ranged from 3.11% to 97.00% and 19.57% to 81.71%, respectively (Table 4). The result indicated that the pyramiding lines with multiple hybrid sterility loci still had the more prominent bridge effect in the BC_1_F_1_ generation.

**Table 4:** Pollen grain fertility of BC_2_F_1_ from the crosses between NILs and two *O. glaberrima* accessions.

Crosses	Average (%)	Standard deviation	Least value (%)	Maximum value (%)	Plant number
DJY1/*O. glaberrima*///DJY1^a^	30.93	21.89	0.00	99.29	516
NIL*S1*/*O. glaberrima*///NIL*S1*	51.01**	34.38	0.00	99.40	1018
NIL*S19*/*O. glaberrima*///NIL*S19*	37.47	23.45	0.00	99.18	65
NIL*S20*/*O. glaberrima*///NIL*S20*	39.53	26.48	0.00	99.40	340
NIL*S37*(t)/*O. glaberrima*///NIL*S37*(t)	31.83	20.69	0.00	98.91	248
NIL*S38*(t)/*O. glaberrima*///NIL*S38*(t)	26.71	27.43	0.00	99.39	152
NIL*S39*(t)/*O. glaberrima*///NIL*S39*(t)	31.23	20.53	0.00	98.51	163
NIL*S1S20*/*O. glaberrima*///NIL*S1S20*	78.92**	26.92	0.00	99.41	663

^a^The symbol “/” indicated the hybridization event, and ”///” indicated the second and third hybridization event with the same male parent.

*Means significant at 0.05 level; ** means significant at 0.01 level.

The average pollen fertility was notably higher in the BC_2_F_1_ generation than those of the BC_1_F_1_ generation. In the crosses between NIL*S1* and the *O. glaberrima* accessions, the average pollen fertility (51.01%) was notable higher than that of the control. The pyramiding lines with two hybrid sterility loci still showed better performance in the BC_2_F_1_ generation. The hybrids between NIL*S1S20* and *O. glaberrima* accessions had the highest average pollen grain fertility (78.92%). It is worth mentioning that the maximum pollen fertility reached normal range (> 95%) in all the hybridization crosses in BC_2_F_1_ generation (Table 4).

## Discussion

Near isogenic lines should be one of the most effective methods on evaluating the HS loci genetic effect and bridge effect. As each NIL had the same DJY1 background and the difference was only on the specific chromosome fragments harboring the specific HS loci, the effect of genetic background could be readily eliminated and the difference between DJY1 and the NILs was originated from the HS locus. Our study showed that the presence of a single HS locus can cause about 50% of gametes sterility. The pollen grain and spikelet fertility of F_1_ plants were semi-sterile when involved *S1* or *S37*(t); however, the pollen grains were semi-sterile and the spikelet fertility was normal when involved *S19*, *S20*, *S38*(t) or *S39*(t). These results indicated that all of the six HS loci followed the “one-locus sporo-gametophytic interaction model”^17^. *S1*, *S37*(t) acted as “gamete eliminator”, while *S19*, *S20*, *S38*(t), and *S39*(t) acted as the “pollen killer”, which is correspond with previous report. Theoretically, the presence of two HS loci can cause about two-thirds of pollen grain abortion and the pollen grain of F_1_ plants would be less than 5% if three HS loci are involved. However, the actual spikelet fertility (50.19%) of F_1_ plants was significantly higher than that of the theoretical value (25%) in the cross of DJY1×NIL*S1S37*(t). The above results indicate that HS loci also involve in inter-loci interaction when they comply with the intra-locus allelic interaction model. Therefore, interspecific hybrid sterility has always been the focus and the difficulty in the rice genetic improvement. The interaction effects between HS loci should be considered in the interspecific hybrid breeding project.

In our case, it was confirmed that some of the “bridge parents” carrying a single HS loci produced interspecific F_1_ progenies with improved pollen fertility. Furthermore, present study showed that the pyramiding lines of multiple HS loci could significantly improve the F_1_ pollen grain fertility than those with single HS locus. In the presence of two HS loci, the pollen grain fertility of hybrid F_1_ increased 5–10 times compared with that of single HS locus when these NILs crossed with *O. glaberrima*. Because the interspecific hybridization involves more HS genes than that of the inter-subspecific hybridization^10^, the “bridge effect” of a single hybrid sterile allele is very limited, even the utilization of pyramiding lines with two or three hybrid sterile alleles cannot obtain ideal "bridge effect". In fact, in the interspecific hybrid breeding project, increasing pollen fertility from 0 to 5% has no remarkable breeding value.

As we known that interspecific hybrid sterility was shown quantitative trait controlled by multiple genes. Although the bridge lines with the *O. glaberrima* alleles can give homozygous genotype at the target loci, other HS loci were still heterozygous in the hybrids, which thus reduced fertility in the F_1_ populations. Backcrossing is an effective method to improve homogeneity of genetic background, and minimize the genetic variation of genetic background. Some of the HS loci become homozygous in the backcross population and as the result the fertility of BC_1_F_1_ plants has significantly improved compared to the F_1_ hybrids. By comparing the bridge effects of HS NILs among F_1_, BC_1_F_1_, and BC_2_F_1_ generations, the BC_1_F_1_ generation showed the most significant bridge effect. Furthermore, the BC_1_F_1_ individuals from the multiple-loci-NILs were notably more fertile than that of the single-locus-NILs*.* Therefore, the optimal solution to improve the fertility of interspecific hybrid can be utilization of pyramiding bridge parent plus backcrossing.

The hybrid sterility also occurs frequently in the inter-subspecific hybridization crosses in rice and the hybrid sterility is mainly affected by five loci involving four for F_1_ male sterility and one for F_1_ female sterility^18^. The *indica*-compatible *japonica* lines (ICJLs) were developed by pyramiding four *indica* allele and one neutral allele in *japonica* genetic background through marker-assisted selection. When the *indica*-compatible *japonica* lines were test-crossed with a set of typical *indica* and *japonica* varieties, the results indicated that the ICJLs were compatible with *indica* while incompatible with *japonica* rice. In the test crosses of the *indica*-compatible *japonica* lines with *indica*, the result showed that the F_1_ pollen and spikelet fertility reversed close to complete fertility when the *indica*-compatible *japonica* lines pyramided with four loci for “pollen killer” and one for “embryo sac killer”^19^. The study showed a great promise of overcoming the intersubspecific hybrid sterility by developing pyramiding lines at HS loci^18^. It can be deduced that the pyramiding lines with *O. glaberrima* alleles on five loci is still not enough on reversing the pollen and spikelet fertility to normal. More efforts are needed to elucidate the effect of various combinations of multiple hybrid sterile bridge loci, and to dissect their interaction or epistatic effect among HS loci.

## Materials and methods

### Developing single-locus-NILs

Five accessions of *O. glaberrima* (Supplementary Table 2) as the donor parents were backcrossed to the Dianjingyou 1 (DJY1), one *O. sativa* ssp. *japonica* variety from Yunnan province, P. R. China. As the result, a series of semi-sterile families of BC_6_F_1_ were obtained in the DJY1 background. In the previous work, six HS locus, *S1*, *S19*, *S20*, *S37*(t), *S38*(t), *S39*(t), for hybrid sterility were identified on chromosome 6, 3, 7, 1, 4, 12, respectively using these BC_6_F_1_ families^14–16^. Based on marker-assisted selection, the plants for the homozygous alleles of *O. glaberrima* on HS loci were obtained from corresponding mapping populations. A whole-genome SNP array (6 k) of rice designed by Cornell University was used to survey the genetic background of the plants with the target HS loci for single-locus-NILs developing^19^. The plants that the genetic backgrounds were similar to the recurrent parent DJY1 were selected as the NILs and designated as NIL*S1*, NIL*S19*, NIL*S20*, NIL*S37*(t), NIL*S38*(t), NIL*S39*(t).

Three NILs, NIL*S1*, NIL*S20* and NIL*S37*(t) were used to develop the pyramiding HS loci lines with molecular marker-assistant method and phenotype selection. The F_2_ self-pollinated plants from the crosses of NIL*S1*/NIL*S37*(t), NIL*S1*/NIL*S20* and NIL*S1*/NIL*S37*(t)//NIL*S20* showing normal pollen and spikelet fertility and homozygous to *O. glaberrima* allele at target loci were selected as the pyramiding lines.

### Evaluation genetic effect and bridge effect of HS loci

Six single-locus-NILs and three multiple-loci-NILs were used as test lines to cross with their recurrent parent DJY1 to evaluate genetic effect of HS loci.

In addition, DJY1 and its nine NILs were used as female and male parents to make reciprocal crosses with two *O. glaberrima* accessions to evaluate the bridge effect of the HS loci. The crosses of *O. glaberrima* with DJY1 were used as the control. Two *O. glaberrima* accessions, IRGC102263 and IRGC103469, from the International Rice Research Institute (IRRI), and *O.sativa* variety DJY1 were used as the tested lines in this study. The F_1_ plants were backcrossed as females to their corresponding NILs untill the BC_2_F_1_ generation was achieved.

All materials were planted at the Winter Breeding Station, YAAS, Sanya, Hainan Province, P. R. China. The first cropping season was from November to April of the following year, and the second cropping season was from July to October.

### Phenotypic evaluation

The pollen grain and spikelet fertility for all parental lines, NILs, F_1_, BC_1_F_1_ and BC_2_F_1_ plants were evaluated. Pollen grain fertility was investigated following the instructions of Zhu^20^. Pollen grain fertility was measured using anthers collected from spikelets at 1 to 2 days before anthesis and stored in 70% ethanol^21^. Three to four anthers per floret per plant were mixed and stained with 1% I-KI solution, and more than 300 pollen grains were observed under a light microscope. Sterile types were further classified as typical, spherical or stained abortion types^22^. Three independent microscopic fields were scored for estimation of the percentage of the four types of pollen grains in each plant. Spikelet fertility was scored as the fertilized spikelet rate of three to five panicles on each plant.

### Molecular marker and assay

The SSR molecular markers linked with *S1*, *S19*, *S20, S37*(t)*, S38*(t)*,* and *S39*(t) were selected on rice microsatellite maps^23^*.* The SSR markers linked with the target HS loci were used for developing the single-locus-NIL, confirming the true hybrid and optimizing the HS loci pyramiding process (Supplementary Table 3). Genomic DNA was extracted from the young leaves of each rice plant following simple DNA extraction method^24^. At least two SSR markers on each HS locus that have polymorphism between DJY1 and NILs were selected. Polymerase chain reaction (PCR) was performed according to McCouch et al.^20^ with minor modifications.

Total 22 plants with the introduced target fragment of *O. glaberrima* were examined using the Cornell_6K_Array_Infinium_Rice (C6AIR) SNP array^19^. Young leaves from each plant and recurrent parent DJY1 were used to isolate genomic DNA using the CTAB mothed. The quality of DNA was checked on 0.8% agarose gels, and the quantity was checked using a Nano-Drop spectrophotometer. The concentration of each DNA sample was adjusted to 50 ng/μl. DNA were used for genotying through the SNP array as described in Thomson et al.^19^. The genotypes of the called SNP were assigned as “A” (DJY1 genotype), “B” (donor parent genotype) and “H” (heterozygous genotype). An unambiguous graphic genotype for each NIL were achieved by R software.

### Statistical analysis

Statistical analysis of the data was performed using one-way ANOVA, and the Student's test was used for further pairwise comparisons if ANOVA differences were significant. Pollen grain and spikelet fertility data as a percentage was transformed by function arcsine square root before the analysis but are listed as percentages.

### Ethics declarations

The plant collection and use was in accordance with all the relevant guidelines.

### Permissions statement

The rice cultivars involved in this paper have permission.

### Supplementary Information

Supplementary Table S1.

Supplementary Table S2.

Supplementary Table S3.

## Data Availability

All relevant data are within the paper.
