# Supplementary material for: Improving bridge effect to overcome interspecific hybrid sterility by pyramiding hybrid sterile loci from Oryza glaberrima
Source: Sci Rep. 2023 Dec 27;13:23057. doi: 10.1038/s41598-023-49914-4 (PMC10754949; doi:10.1038/s41598-023-49914-4)
Supplement: Supplementary file 2 — Supplementary Table S2. [file 41598_2023_49914_MOESM2_ESM.docx]

| Table S2 The information of six single-locus-NILs | | |
| --- | --- | --- |
| HS loci | crosses | donor |
| *S1* | IRGC103145/DJY1/8/DJY1 | IRGC103145 |
| *S19* | IRGC103146/DJY1/8/DJY1 | IRGC103146 |
| *S20* | DJY1/9/IRGC101854/8/DJY1 | IRGC101854 |
| *S37(t)* | IRGC102375/DJY1/8/DJY1 | IRGC102375 |
| *S38(t)* | IRGC103466/DJY1/8/DJY1 | IRGC103466 |
| *S39(t)* | DJY1/9/IRGC101854/8/DJY1 | IRGC101854 |
