# Supplementary material for: Improving bridge effect to overcome interspecific hybrid sterility by pyramiding hybrid sterile loci from Oryza glaberrima
Source: Sci Rep. 2023 Dec 27;13:23057. doi: 10.1038/s41598-023-49914-4 (PMC10754949; doi:10.1038/s41598-023-49914-4)
Supplement: Supplementary file 3 — Supplementary Table S3. [file 41598_2023_49914_MOESM3_ESM.docx]

| Table S3 The SSR marker used to develop the HS NILs | | | | |
| --- | --- | --- | --- | --- |
| Marker name | chro. | linked with HS loci | Forward primer | Reverse primer |
| RM190 | 6 | *S1* | ctttgtctatctcaagacac | ttgcagatgttcttcctgatg |
| RM587 | 6 | *S1* | acgcgaacaaattaacagcc | ctttgctaccagtagatccagc |
| RM3372 | 3 | *S19* | GAGCGACCAAAGAATCCAAG | CCACGGGGAGCTGATGAAG |
| RM22 | 3 | *S19* | ggtttgggagcccataatct | ctgggcttctttcactcgtc |
| RM20847 | 7 | *S20* | CTCGATCATCCGTCTTATATCTGG | GGGATTATTCTTGCCTTGATGG |
| RM20852 | 7 | *S20* | GTAGCTCCATGCCAGTTTGTGG | AACCTTCTTGATTGGCCATCTCC |
| RM449 | 1 | *S37(t)* | ccttgaccctcctcttactt | tcctatcaaaatttggcaac |
| RM513 | 1 | *S37(t)* | tctagtggcctcaaaaaggg | gcaacgaaatcatccctagc |
| RM16260 | 4 | *S38(t)* | TGCTAGTAGGAGAGGAGCGAAGC | ACTGTGAGTCGTGTCAGAGTTGG |
| RM5414 | 4 | *S38(t)* | ACCATGGTTCAAGAGTGAAA | ACAGCTCAACCTGTTGAGTG |
| RM1880 | 12 | *S39(t)* | ACCACTAAATAAGCACATAC | GGCATCATACATTAAAATAC |
| RM4 | 12 | *S39(t)* | ttgacgaggtcagcactgac | agggtgtatccgactcatcg |
|  |  |  |  |  |
